# [Corrigendum] Circular RNAs in osteosarcoma: An update of recent studies (Review)

**DOI:** 10.3892/ijo.2024.5696

**Published:** 2024-09-23

**Authors:** Le Zeng, Longzhou Liu, Wen-Juan Ni, Fuhua Xie, Xiao-Min Leng

Int J Oncol 63: 123, 2023; DOI: 10.3892/ijo.2023.5571

Subsequently to the publication of the above review, the authors have contacted the Editorial Office to explain that the article was regrettably published containing a few errors. First, on p. 3, left-hand column, the '**3. Functions of circRNAs in OS**' section, line 23, the sentence here should have read as follows (changes shown in bold, where appropriate): '**Circ_0001649** has been reported as **a sponge** of various miRNAs that inhibit cell proliferation (62,83).' (i.e., mentioning 'Circ_0001649' twice was an error/oversight). Secondly, in the '**4. Mechanisms of circRNAs in OS**' section, paragraph 5, line 23 on p. 8, the four consecutive sentences that start on this line should have read as follows: '**Hsa_circ_0000190** is significantly downregulated in OS tissues and cell lines. This circRNA inhibits the Wnt/β-catenin signaling pathway by sponging miR-767-5p, the target of which is TET1 (**61)**. And hsa_circ_0002052 can sponge miR-1205, the target of which is adenomatosis polyposis coli 2 (APC2), a negative regulator of the Wnt/β-catenin signaling pathway. Hence, **hsa_circ_0000190 and hsa_circ_0002052 can function** as inhibitors of the Wnt/β-catenin signaling pathway by promoting TET1 and APC2 expression via miRNA sponging, ultimately resulting in the delayed development of OS **(50,61)**.' (i.e., the first sentence was corrected to read 'Hsa_circ_0000190' and 'hsa_circ_0000190' was added to the fourth sentence, and ref. 61 was added to the second sentence in this section, and included with ref. 50 at the end of the fourth sentence). Thirdly (and finally), changes were required to both Fig. 3 and its accompanying legend, and these are featured on the next page; essentially, 'CircRNA CDR1as (47)' should not have been included in the Fig. 3 legend as this circRNA is not described in the figure, and some changes have been made to the figure itself in terms of wrongly placed lines and arrows.

The authors are grateful to the Editor of *International Journal of Oncology* for allowing them this opportunity to publish this Corrigendum, and all the authors agree with its publication. Furthermore, the authors apologize to the readership for any inconvenience caused.

## Figures and Tables

**Figure 3:**
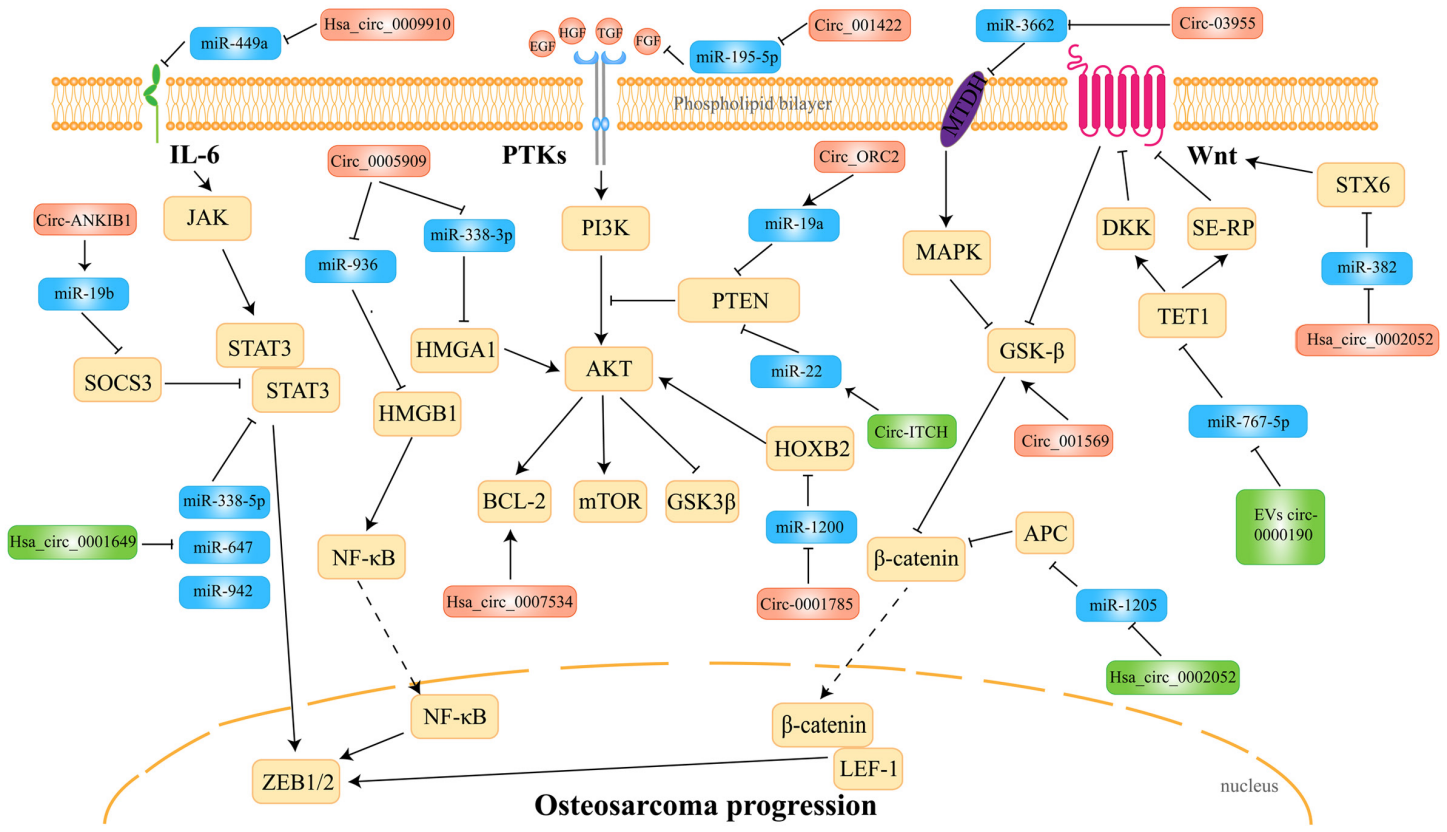
Mechanisms of circRNAs in OS. Hsa_circ_0007534 (46), Circ_ORC2 (51), Circ_001422 (63), Circ_0001785 (52), Hsa_circ_0005909 (64), Circ_03955 (54), Circ_001569 (48), Hsa_circ_0002052 (56), Hsa_circ_0009910 (49), Circ_ANKIB1 (57), Circ_ITCH (53), Hsa_circ_0005909 (55), Hsa_circ_0002052 (50), Has_circ_0000190 (61), Hsa_circ_0001649 (62) participate in the occurrence and development of OS by regulating the JAK-STAT3, NF-κB, PI3K-AKT and Wnt/β-catenin signaling pathways. SOCS3, suppressor of cytokine signaling 3; STAT3, signal transducer and activator of transcription 3; HMGA1, high mobility group AT-hook 1; HMGB1, high mobility group box 1; ZEB1/2, zinc finger E-box binding homeobox 1/2; PTEN, phosphatase and tensin homolog; GSK3β, glycogen synthase kinase 3β; HOXB2, homeobox B2; DKK, Dickkopf; TET1, Tet methylcytosine dioxygenase 1; SFRP2, secreted frizzled-related protein 2; STX6, syntaxin 6.

